# Two Instances of the Effects of Drinking Pure Spirits, in Repeated and Large Quantities

**Published:** 1786

**Authors:** John Rollo

**Affiliations:** late Surgeon in the Royal Artillery


VII. *Two Inſtances of the Effects of drinking pure Spirits, in repeated and large Quantities. Communicated in a Letter to Dr.* Simmons, *F. R. S. by* John Rollo, *M. D. late Surgeon in the Royal Artillery.*

THE following caſes will ſerve to ſhow the effects of drinking pure ſpirits, in repeated and large quantities. The firſt is the caſe of a man of an athletic conſtitution; the ſecond that of a patient whoſe bowels had been previouſly debilitated by diſeaſe. The laſt was communicated to me by Mr. Cruikſhanks, an ingenious ſurgeon of the Naval Hoſpital, in Barbadoes. I had occaſion to mention another caſe of the ſame kind, in my obſervations on the means of preſerving and reſtoring health, in the Weſt Indies. Caſes of this ſort may be deemed rare occurrences; but it ſeems probable probable that ſimilar effects are not unfrequently, though more imperceptibly produced by ſpirits drank in ſmaller quantities, but uniformly from day to day repeated, by various and numerous deſcriptions of people. The caſes and diſſections, therefore, may perhaps not be deemed unworthy of your notice.

### CASE I.

Thomas Speachly, aged 34 years, was by trade a blackſmith, and had been a matroſs in the artillery three or four years. Soon after his arrival from England, at Barbadoes, he was employed as an artificer in the blackſmith's ſhop. He addicted himſelf to the uſe of rum, in large quantities, at all hours, and without mixture. He has been known to remain in the workſhop, two or three days without any ſubſiſtence, ſave rum. He appeared generally ſtupid, with marks of intoxication. This courſe of life produced a very irritable ſtate of the ſtomach, which at length rejected every thing he ſwallowed, by an immediate vomiting.

At this period, being nearly five weeks preceding his death, a looſeneſs, parched tongue, great great thirſt, and a quick, feeble pulſe ſupervened.

He had at intervals, an abatement of the vomiting, and for ſeveral days his belly was tolerably regular. A variety of medicines were exhibited without any apparent good effect. Some advantage was derived from a bliſter applied over the whole extent of the epigaſtric region. Opium in ſeveral forms gave little or no relief; and without any material alteration in his complaint he gradually lingered and died.

The contents of the thorax were in a natural ſtate, except an adheſion of the right lobe of the lungs to the pleura. In the abdomen, diſeaſe was evidently marked. The whole of the viſcera had a reddiſh appearance. The ſtomach was diminiſhed in ſize, and its inner coat almoſt entirely deſtroyed, except a ſmall part which had ulcerations on it. The ſtomach contained no gaſtric juice, or that ſlimy fluid uſually found in it. Its contents were about half a pint of a thick and black-coloured liquor, reſembling the bile we found in the gall bladder. The gall bladder was much diſtended with bile of a viſcid conſiſtence, and of a dark colour. The interior coat of all the inteſtines had a reddiſh appearance. The liver was was larger than common, but in other refpects as it is naturally. The fpleen was of three times its natural fize, and it adhered very firmly at one part to the liver. The pancreas was fchirrous. The urinary bladder contained a quantity of very thick urine.

### CASE II.

John Hayes, aged thirty years, was admitted into the Naval Hofpital in Barbadoes, with fcurvy. As this difeafe began to difappear, he had a loofe ftate of his belly, with griping, but he gradually got better.

About fix weeks after his recovery he drank, for feveral days, a great quantity of pure rum. He was then feized with vomiting, violent gripings, and frequent whitifh ftools, ftreaked with blood. Thefe fymptoms continued three weeks without much alteration.

Five days before his death, the ftools were more flimy, but without blood; they ran off involuntarily: at this time he became delirious.

On opening the body, the liver was found to be much enlarged, and the gall bladder much diftended with bile of a light green colour. The ftomach was contracted, and inflamed flamed throughout its whole internal furface. The duodenum was alfo inflamed, though flightly. The jejunum was of a natural colour. The ileum had its internal coat confiderably inflamed. The beginning of the colon was of a natural appearance; but from the middle of the tranfverfe arch, all the way to the anus, there was an uniform appearance of inflammation, of a dark hue. In the rectum there were fome fmall ulcerations very near to the anus, but no where elfe. In this cafe the appearance of inflammation was more remarkable through the whole tract of the inteftines, than I ever had feen it before.

It would be fuperfluous to add any particular remarks, as thefe will naturally arife fo far as they can be ufeful to every intelligent practitioner who perufes the cafes, and who may occafionally meet with fimilar fymptoms of difeafe among dram drinkers. It may, however, not be unneceffary to obferve, that any advantage from medicine in fuch cafes, is only to be expected from the ufe of blifters, warm fomentations, and, under proper circumftances, repeated blood-letting.

*Woolwich,*
Nov. 7th 1785.

